# Papillary Fibroelastoma of Aortic Valve: An Echocardiographic Correlation With Surgery and Anatomopathology

**DOI:** 10.1016/j.jaccas.2024.102842

**Published:** 2024-12-18

**Authors:** Jorge Mandiola, Felipe Castañia

**Affiliations:** a Laboratory of Echocardiography, Hospital Clínico Regional de Antofagasta, School of Medicine University of Antofagasta, Antofagasta, Chile; b Department of Cardiology, Valdivia Hospital, School of Medicine Austral University, Valdivia, Chile

**Keywords:** aortic valve, echocardiography, papillary fibroelastoma

## Abstract

This case presents a 69-year-old woman with a previous history of arterial hypertension. A transthoracic echocardiogram was requested in the context of shortness of breath with great exercise. Incidentally, at the aortic valve level, a mobile mass suggestive of papillary fibroelastoma was visualized. Therefore, a complementary transoesophageal echocardiogram was performed.

In this patient, because the size lesion is above 10 mm, mobile, and located in the aortic valve, with a low perioperative surgical risk, a decision to perform cardiac surgery was made. A preoperative coronarography showed a significant proximal descending anterior artery lesion. Finally, the patient was taken to the operative room, where a coronary artery bypass graft surgery on pump with a left internal mammary artery to descending anterior artery and successful excision of papillary fibroelastoma from the left coronary cusp were realized (Figure 1), with favorable postoperative evolution. The patient was discharged without drawbacks.

**Take-Home Message**

• A very good echocardiography is pivotal to make the difference in PFE diagnosis, and 3D imaging further enhances the correlation with anatomopathology.

**Figure 1:**
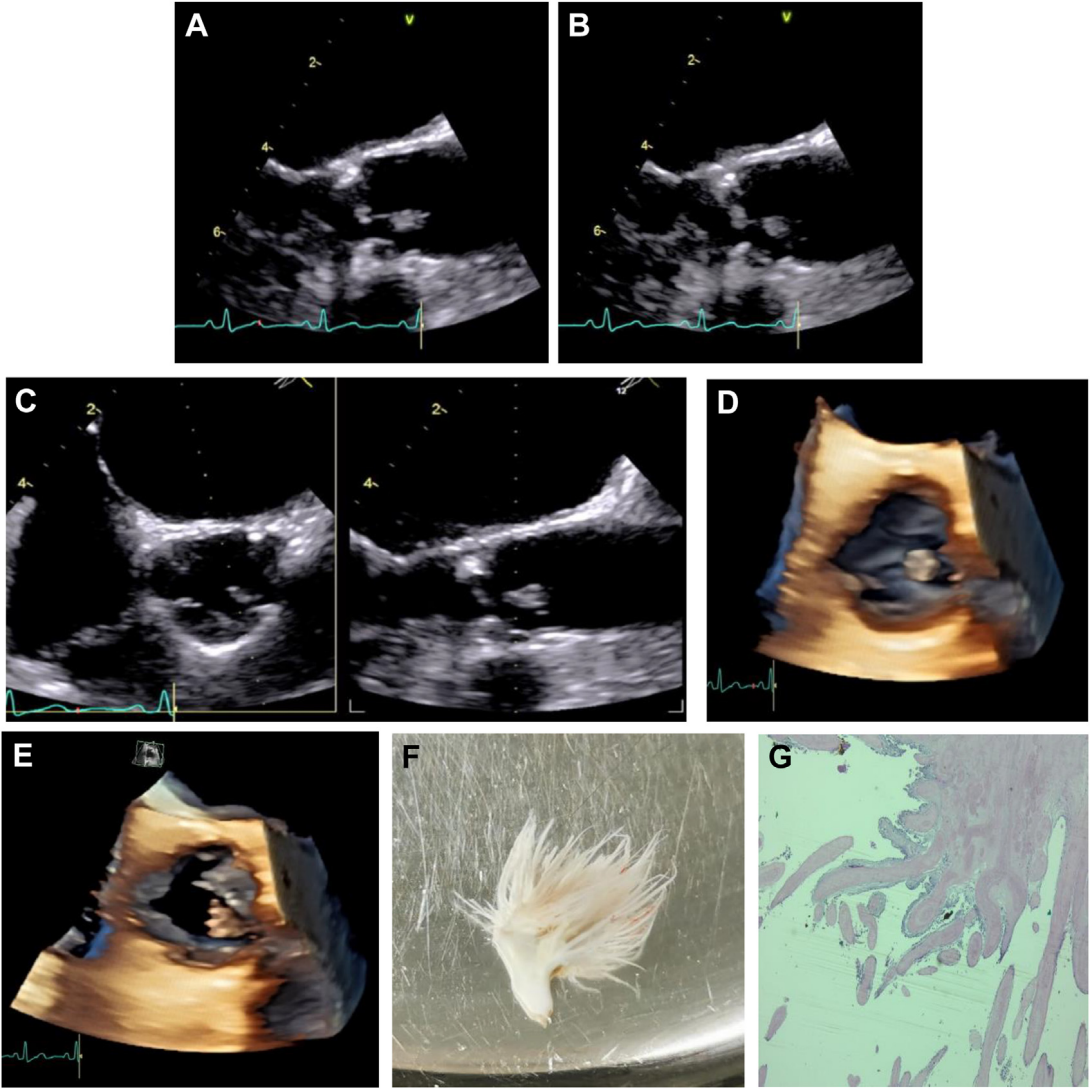
Papillary Fibroelastoma of Aortic Valve: an Echocardiographic Correlation With Surgery and Anatomopathology (A and B) Aortic valve zoom at 141°: at the aortic face and free edge of the left coronary cusp, a heterogeneous mass of 12 × 4 mm with irregular edges, mobile and flaming, pedicled with multiple terminal digitiform branches that resemble a sea anemone was visualized, which very probably corresponds to a papillary fibroelastoma (PFE) (Video 1). (C) Orthogonal multiplane image (50° and 140°) aortic valve (Video 2). (D and E) Zoom 3-dimensional image of the aortic valve (Video 3) (HD Live, Vivid E95; GE HealthCare). (F) Macroscopic photo of surgical piece and (E) histology with hematoxylin and eosin staining on 100× field, where a papillary architecture lesion with multiple ramifications, avascular fibroelastic connective tissue, and paucicellular center covered by a single layer of endocardium is evident, compatible with PFE.

The papillary fibroelastoma corresponds to the second most frequent cause, behind atrial myxoma, of primary cardiac benign tumor (15% in total). Its prevalence is infrequent (around 0.03%-0.3%), predominantly affecting people between 50 and 80 years of age, with a slight male sex preference. Its etiopathogenesis is not clear, but some authors suggest that it is acquired. It can be unique or multiple and generally affect left cardiac valves; however, it may be in any place in the endocardium. Habitually, they are asymptomatic, but they can manifest as embolic phenomena, stroke, and in very rare cases, myocardial infraction and sudden death. Echocardiography is the fundamental pillar for the diagnosis, where a sea anemone form is characteristic.^1^^,^^2^ The decision to perform a surgery is not completely defined, but experts’ opinions recommend it in asymptomatic cases when the size is above 10 mm, mobile, in left cardiac valves with a low surgical mortality risk (Society of Thoracic Surgeons score <1%), and in symptomatic patients that have presented a cardioembolic event.^3^

## Funding Support and Author Disclosures

The authors have reported that they have no relationships relevant to the contents of this paper to disclose.

## Appendix

Video 1 Zoom 2-Dimensional Aortic Valve A papillary fibroelastoma is visualized at the aortic face and free edge of the left coronary cusp.

Video 2 Multiplane 2-Dimensional Aortic Valve A papillary fibroelastoma is visualized at the aortic face and free edge of the left coronary cusp.

Video 3 Zoom 3-Dimensional Aortic Valve A papillary fibroelastoma is visualized at the aortic face and free edge of the left coronary cusp.
